# Antibacterial resistance and the success of tailored triple therapy in *Helicobacter pylori* strains isolated from Slovenian children

**DOI:** 10.1111/hel.12400

**Published:** 2017-06-27

**Authors:** Tita Butenko, Samo Jeverica, Rok Orel, Matjaž Homan

**Affiliations:** ^1^ Department of Gastroenterology, Hepatology, and Nutrition University Children's Hospital Ljubljana Slovenia; ^2^ Faculty of Medicine Institute of Microbiology and Immunology University of Ljubljana Ljubljana Slovenia

**Keywords:** children, eradication rate, infection with *Helicobacter pylori*, primary resistance

## Abstract

**Background:**

Primary *Helicobacter pylori* (*H. pylori*) infection occurs predominantly in childhood. Antimicrobial resistance is the leading cause for *H. pylori* eradication failure. The aims of this study were (i) to establish for the first time the antimicrobial resistance of *H. pylori* strains in infected Slovenian children not previously treated for *H. pylori* infection and (ii) to evaluate the effectiveness of tailored triple therapy, assuming that eradication rate with tailored triple therapy will be >90%.

**Methods:**

Data on all treatment‐naive children 1‐18 years old and treated for *H. pylori* infection according to susceptibility testing were retrospectively analyzed. All relevant clinical information and demographical information were retrospectively collected from the hospital information systems and/or patients’ medical documentation.

**Results:**

The inclusion criteria were met by 107 children (64.5% girls) with a median age of 12.0 years (range 2.0‐17.6 years). Primary antimicrobial resistance rates of *H. pylori* were 1.0% to amoxicillin (AMO), 23.4% to clarithromycin (CLA), 20.2% to metronidazole (MET), 2.8% to levofloxacin (LEV), and 0.0% to tetracycline (TET). Dual resistances were detected to CLA and MET in 11.5% (n=12) of strains, to CLA and LEV in 2.8% (n=3), and to MET and LEV in 2.9% (n=3). Results of treatment success were available for 71 patients (66.2% girls). Eradication of *H. pylori* was evaluated using the 13C‐urea breath test, monoclonal stool antigen test or in some cases with repeated upper GI endoscopy with histology and cultivation/molecular tests. Eradication was achieved in 61 of 71 (85.9%) patients.

**Conclusions:**

The primary resistance rates of *H. pylori* to CLA and MET in Slovenia are high. Our data strongly support the fact that in countries with high prevalence of resistant *H. pylori* strains susceptibility testing and tailored therapy is essential.

## 1 INTRODUCTION

Infection with *H. pylori* is still the most common bacterial infection in humans, despite the significant worldwide decrease in its incidence and prevalence recorded in recent years.^1^ Chronic inflammation of the gastric mucosa can cause peptic ulcers, gastric erosions, and mucosa‐associated lymphoid tissue lymphoma in children. In addition, extra gastric complications, such as iron‐deficiency anemia, may be induced by *H. pylori* infection.^2^ The first‐line treatments currently available for children infected with *H. pylori* are various combinations of proton‐pump inhibitors (PPI) with antibiotics including AMO, macrolides, and/or imidazoles.^3^, ^4^ In the last decade, the eradication rates using these schemes have declined and have led to recommendations for higher dosages and longer duration of therapy or alternative regimens, including three instead of two different antibiotics, either sequentially or concomitantly.^4^, ^5^ The low success rates are essentially due to the increasing resistance rates to macrolides and to a lesser extent imidazoles. Koletzko et al.^6^ detected very high primary resistance rates to metronidazole (MET) and clarithromycin (CLA) in Europe (23% and 20%, respectively), and resistance after failed eradication therapy was even higher (35% and 42%, respectively). Children born to mothers from developing countries have extremely high primary resistance rates to MET presumably due to the frequent use of this drug for parasitic infections in Africa and Asia. Higher primary CLA resistance rates in pediatric patients compared to adults are thought to be a consequence of frequent use of macrolides for treatment of respiratory infections. Therefore, in order to improve eradication rates in children infected with *H. pylori*, the latest ESPGHAN and NASPGHAN guidelines for developed countries suggest eradication therapy according to the results of antimicrobial susceptibility testing. Namely, antibiotic susceptibility testing for CLA is recommended before initiation of CLA‐based triple therapy in areas/populations with a known high resistance rate (>20%) of *H. pylori* to CLA or where antibiotic resistance rates are unknown.^3^

The aims of this study were (i) to establish for the first time the antimicrobial resistance of *H. pylori* strains among Slovenian children not previously treated for *H. pylori* infection and (ii) to evaluate the effectiveness of tailored triple therapy, assuming that eradication rate with tailored triple therapy will be >90%.^7^

## 2 METHODS

### 2.1 Study design and patients

We retrospectively analyzed data for all treatment‐naive children 1‐18 years old that were treated for *H. pylori* infection according to antimicrobial susceptibility results from 2011 to 2014. Four Slovenian hospitals were involved in this multicenter retrospective cohort study: Celje General Hospital, Ptuj General Hospital, Slovenj Gradec General Hospital, and Ljubljana University Children's Hospital. Basic demographic and clinical characteristics were extracted from the hospital information systems and/or patients’ medical documentation. Data included sex, age at diagnosis, endoscopy findings, rapid urease test (RUT) results, histology findings, microbiological culture with antimicrobial susceptibility results, treatment composition and duration, side effects, and eradication rate. Patients were diagnosed for *H. pylori* infection in accordance with the latest ESPGHAN and NASPGHAN guidelines, which recommend that initial diagnosis of *H. pylori* infection is based on either positive histopathology and a positive RUT or a positive culture.^3^ The success of eradication was evaluated with the 13C‐urea breath test (UBT) or monoclonal stool antigen test at least 4 weeks after the treatment. Previous eradication therapy, the use of PPI, anti‐secretory drugs, or antimicrobial agents 1 month prior to the upper endoscopy, or an eradication protocol not in accordance with antimicrobial susceptibility results was exclusion criteria.

The study was approved by the Republic of Slovenia National Medical Ethics Committee, no. 153/06/14.

### 2.2 Biopsy sampling and antimicrobial susceptibility testing

Biopsies were taken during upper endoscopy from the duodenum, gastric antrum and corpus, and distal esophagus for histology; additional biopsies were taken from the antrum for RUT and cultivation. The antral biopsies were collected and transported to the Institute of Microbiology and Immunology, Faculty of Medicine, University of Ljubljana. Biopsy specimens were homogenized and plated on three agar media: Pylori agar (bioMérieux, Marcy l'Etoile, France) and two in‐house agar media enriched with 9% horse serum and 9% human blood and incubated for 3‐9 days at 37°C in a microaerophilic atmosphere. Identification of *H. pylori* was confirmed using typical colony morphology, urease, catalase, and oxidase tests. Culture‐positive biopsy specimens were phenotypically tested for susceptibility to AMO, CLA, MET, levofloxacin (LEV), and tetracycline (TET) using the gradient‐diffusion method, ISO Sensitest agar supplemented with defibrinated horse blood, and EUCAST breakpoints tables version 4.0 for interpretation criteria based on epidemiological cutoff values, which distinguish wild‐type isolates from those with reduced susceptibility. Strains were resistant to antimicrobial agent when minimal inhibitory concentrations were >0.125 mg/L for AMO, >0.5 mg/L for CLA, >8 mg/L for MET, and >1 mg/L for LEV and TET. All culture‐negative specimens were further tested for susceptibility to CLA using two molecular assays: the in‐house real‐time PCR targeting well‐described 23S rRNA mutations and the Genotype HelicoDR (Hain Lifescience GmbH, Nehren, Germany) commercial assay with the same target sites.

### 2.3 Tailored triple therapy

A 7‐ to 14‐day treatment was tailored according to the results of antimicrobial susceptibility results of strains isolated from gastric biopsies of the individual patient. It was comprised of a PPI and two antimicrobial agents to which the *H. pylori* strain was susceptible. Eradication therapy included AMO (50 mg kg^−1^ day^−1^ in two doses, maximum 2 g/day)+MET (20 mg kg^−1^ day^−1^ b.i.d, to a maximum 800 mg/day) or CLA (15 mg kg^−1^ day^−1^ b.i.d., maximum 1 g/day. The dosage used for PPI was 1 mg kg^−1^ day^−1^, b.i.d. maximum 40 mg.

### 2.4 Follow‐up to assess the success of guided triple therapy

The eradication rate was evaluated using the UBT, a stool antigen test, or in some cases repeated upper GI endoscopy with histology and cultivation/molecular tests 8 weeks after the tailored therapy. The cutoff value of the UBT used (Helicobacter test INFAI, INFAI UK Ltd., York, UK) was 4‰. According to the manufacturer's instructions, the results of the stool antigen test were defined as negative, positive, or indeterminate. Because the results of the UBT and stool antigen test were often reported only to the child's primary pediatrician and not to the pediatric gastroenterologist that prescribed the eradication therapy, we inquired about those results in writing or with a phone call.

### 2.5 Statistical analysis

The results were collected into an electronic database (Excel, Microsoft, USA). Statistical analysis was performed using IBM SPSS ver. 21 (IBM Corporation, Armonk, NY, USA). Categorical variables were summarized as frequencies and percentages; the median and range were calculated for the patient's age. Univariate logistic regression was performed with *H. pylori* eradication as a dependent variable and sex, age groups (1‐11 or 12‐18 years old), duration of antimicrobial therapy, and therapy with CLA or MET as covariates. Odds ratios (ORs) and 95% confidence intervals were calculated. A *P*‐value <.05 indicated significant differences.

## 3 RESULTS

### 3.1 Population characteristics

During the study period, we identified 107 treatment‐naive children eligible for *H. pylori* eradication therapy; 64.5% (n=69) were girls and 35.5% (n=38) boys. The median age was 12.0 years (range: 2.0‐17.6 years). Of them, 43.0% (n=46) belonged to the age group 1‐11 years old, and 57.0% (n=61) to the age group 12‐18 years old. Nodular gastritis was the predominant endoscopic finding present in 98.1% (n=105) of patients, erosive gastritis was present in 14.0% (n=15), 3.7% (n=3) of patients had a gastric ulcer, and 0.9% (n=1) had a duodenal ulcer. The population characteristics and endoscopic data are summarized in Table 1.

**Table 1:** Basic demographic characteristics, age, distribution, and endoscopic findings

Characteristics	n (%)
Patients	107 (100.0)
Girls	69 (64.5)
Boys	38 (35.5)
Age distribution (y; median=12.0, range=2.0‐17.6)	
1‐11	46 (43.0)
12‐18	61 (57.0)
Endoscopy findings	
Nodular gastritis	105 (98.1)
Erosive gastritis	15 (14.0)
Gastric ulcer	3 (3.7)
Duodenal ulcer	1 (0.9)

### 3.2 Antimicrobial resistance of *Helicobacter pylori*

Altogether, 107 strains from treatment‐naive children were tested for antimicrobial susceptibility, of which 104 were culture‐positive and were consequently tested with phenotypic methods. Three strains were culture‐negative. For these three strains, susceptibility testing was performed with genotypic methods, and only results for CLA and LEV were available. Primary resistance rates were 23.4% (n=25) to CLA, 20.2% (n=21) to MET, 2.8% (n=3) to LEV, 1.0% (n=1) to AMO, and 0.0% (n=0) to TET. Dual resistance was detected to CLA and MET in 11.5% (n=12) of strains, to CLA and LEV in 2.8% (n=3), and to MET and LEV in 2.9% (n=3). Triple resistance to CLA, MET, and LEV was detected in 2.9% (n=3) of strains, and quadruple resistance to CLA, MET, LEV, and AMO in 1.0% (n=1). Approximately 68.3% (n=71) of *H. pylori* strains were sensitive to all antimicrobial agents tested. The data are shown in Table 2.

**Table 2:** Primary antimicrobial resistance of *Helicobacter pylori* from Slovenian children, 2011‐2014

Antimicrobial agent	Strains tested	Primary antibiotic resistance
	N	n (%)
Overall resistance to antimicrobial agent		
Clarithromycin (CLA)	107	25 (23.4)
Metronidazole (MET)	104	21 (20.2)
Levofloxacin (LEV)	107	3 (2.8)
Amoxicillin	104	1 (1.0)
Tetracycline	104	0 (0.0)
Resistance to >1 antimicrobial agent		
CLA, MET	104	12 (11.5)
CLA, LEV	107	3 (2.8)
MET, LEV	104	3 (2.9)
CLA, MET, LEV	104	3 (2.9)
CLA, MET, LEV, amoxicillin	104	1 (1.0)

### 3.3 Antimicrobial therapy eradication rate

Inclusion criteria for evaluation of antimicrobial therapy were met in 66.4% (n=71) children (per‐protocol population). Among the excluded patients, 15.0% (n=16) did not respond to the questionnaire, 6.5% (n=7) did not perform evaluation of therapy with the UBT or stool antigen test, 6.5% (n=7) did not receive therapy according to antimicrobial susceptibility results, and 5.6% (n=6) were lost to follow‐up for other reasons.

The duration of therapy was known for 95.8% (n=68) patients. The majority, 73.5% (n=50) received 14 days of therapy, 16.2% (n=11) received 7 days, and 10.3% (n=7) received 10 days. Regarding antimicrobial agents used for eradication treatment, 66.2% (n=47) were treated with AMO and CLA, 22.5% (n=16) with AMO and MET, 4.2% (n=3) with CLA and MET, 4.2% (n=3) with AMO and LEV, and 2.8% (n=2) with AMO and TET. Overall, eradication was achieved in 85.9% (n=61) of children. Using univariate logistic regression, there was no statistical significant difference in eradication with regard to sex, age group, treatment duration, and antimicrobial therapy using CLA or MET (*P*>.05). The eradication rates are shown in Table 3.

**Table 3:** Eradication rate of triple tailored therapy among treatment‐naive pediatric patients in Slovenia, 2011‐2014

Parameters	Eradication		Univariate logistic regression	
	Success	Failure		
	n (%)	n (%)	OR (95% CI)	*P*‐value
Number of patients	61 (85.9)	10 (14.1)		
Gender (n=71)				
Male	20 (83.3)	4 (16.7)	1 (ref)	
Female	41 (87.2)	6 (12.8)	1.37 (0.35‐5.40)	.66
Age groups (n=71)				
1‐11 y	20 (80.0)	5 (20.0)	1 (ref)	
12‐18 y	41 (89.1)	5 (10.9)	2.05 (0.53‐7.91)	.30
Duration of treatment (n=68)				
7 or 10 d	16 (88.9)	2 (11.1)	1 (ref)	
14 d	42 (84.0)	8 (16.0)	0.66 (0.13‐3.43)	.62
Antimicrobial therapy				
Without Clarithromycin (CLA)	18 (85.7)	3 (14.3)	1 (ref)	
With CLA	43 (86.0)	7 (14.0)	0.98 (0.23‐4.21)	.98
Without metronidazole (MET)	43 (17.3)	9 (82.7)	1 (ref)	
With MET	18 (94.7)	1 (5.3)	3.77 (0.44‐31.96)	.22

Adverse effects were present in 21.2% (n=15) of patients. These included nausea in 14.1% (n=10) of patients, abdominal pain in 7.0% (n=5), vomiting in 7.0% (n=5), and taste disturbance in 2.8% (n=2). None of the patients stopped their therapy due to adverse effects.

## 4 DISCUSSION

Continuous surveillance of both antimicrobial resistance of *H. pylori* as well as different treatment modalities’ success rate is crucial for improving and developing optimal treatment. CLA‐based therapy is still the mainstay for *H. pylori* eradication. However, the pediatric population is prone to development of CLA resistance due to a high burden of respiratory tract infections, for which macrolide antibiotics are often prescribed. For this reason, some suggest that CLA should not be used for *H. pylori* eradication unless susceptibility has been confirmed.

This is the first study that evaluated the success rate of *H. pylori* eradication in Slovenian children. Our study detected high primary resistance rates for both CLA and MET and substantial dual or multidrug resistance rate (Table 2). The eradication rate with tailored therapy was 85.9% and did not achieve the target rate of >90%. However, the latter target was set based on dual resistance rate of <15%^7^ to which our dual resistance rate came fairly close at 11.5%. Therefore, the below expected eradication rate might have been expected.

The first aim of this multicenter study was to establish the primary antimicrobial resistance of *H. pylori* among Slovenian children for the first time. Our study showed a high proportion of CLA resistance (23.4%). These results are similar to results from a large prospective multicenter study published by Koletzko et al.^6^ that included 17 centers from 14 different European countries and revealed 20% resistance to CLA among European children. Primary resistance of *H. pylori* to CLA was also recently investigated among Portuguese and Austrian children. The authors of the Portuguese study found 35% resistance to CLA,^8^ whereas resistance among the Austrian children was 34%.^9^ The Austrian study also showed a significant increase in primary resistance to CLA compared to the years before 2000. The authors concluded that frequent use of CLA in Austria could be the main reason for this increase. This is supported by a study performed by Boyanova et al. in children in Bulgaria. The resistance of *H. pylori* to CLA in their study was 12% and they attributed this to relatively low macrolide consumption in Bulgaria.^10^ The lowest primary CLA resistance (1%‐5%) was reported in the Netherlands.^11^, ^12^ Janssen et al.^11^believe that these differences in primary resistance to CLA in the Netherlands compared to other European countries may be related to the extremely low use of antibiotics. We believe that the reason for the lower incidence of CLA resistance in Slovenia in comparison with neighboring countries could be due to the activities introduced by the National Institute of Public Health of Slovenia in 1999 to reduce antibiotic consumption. A recent study by Fürst et al.^13^ showed that macrolide utilization in Slovenia halved between 1999 and 2012. However, because there are no studies on antimicrobial resistance of *H. pylori* among Slovenian children before that period, it is impossible to assess time trends in resistance and to correlate them with antimicrobial consumption.

Several studies on the antimicrobial resistance of *H. pylori* among European children have shown primary resistance to MET at 10%‐35%, to AMO at 0%‐1%, and to LEV at 0.2%‐0.6%,^6^, ^9^, ^10^, ^14^, ^15^ whereas resistance to TET has not generally been reported in Europe.^14^ Our results for AMO, MET, and TET are in line with these results, whereas resistance to LEV (2.8%) was slightly higher, but we do not have a good explanation for the difference in results compared to neighboring countries.

Dual resistance to CLA and MET in treatment‐naive patients was reported in up to 17% of children in recent reports.^6^, ^9^, ^15^, ^16^ In our study, an 11.5% (n=12) dual resistance rate was detected, which is in line with the published data.

When evaluating the eradication rate of tailored triple therapy according to susceptibility testing among children in Slovenia, the eradication rate did not achieve 90% as a goal.^7^ The overall eradication rate in this study was 85.9%. It was lower in comparison with previously published results with a >90% and even >95% eradication rate.^17^, ^18^, ^19^ Various factors can be involved in the lower success of treatment in our study, such as antibiotic resistance, mixed infections, and the duration of therapy. Furthermore, our study was retrospective; therefore, we were unable to ensure that all the patients took their eradication therapy as prescribed.

It is well known that in vitro antimicrobial susceptibility does not always correspond to successful eradication in vivo.^20^ Furthermore, antibiotic resistance acquired during the treatment could also be important for treatment failure. Gosciniak et al. studied resistance to MET, CLA, and AMO using *H. pylori* isolates from children before and after treatment. The rate of MET and CLA resistance before treatment was 35% and 9%, and 6 weeks after treatment it was 48% and 18%, respectively.^21^ A similar observation was made by Miyaji et al.,^22^ who concluded that the failure of treatment could be a consequence of the induction of resistance to CLA and MET. Infection with multiple strains of *H. pylori* can also influence eradication success. Yakoob et al. investigated mixed infections among Chinese patients. They obtained biopsy specimens for culture of *H. pylori* from the gastric antrum, corpus, and fundus. *H. pylori* were identified by culture from two or more sites in 10 of the 16 patients. The DNA diversity of the isolates was determined by arbitrarily primed polymerase chain reaction fingerprinting. Of 10 patients with multiple isolates, 70% (seven of 10) exhibited variation in susceptibility to MET and 20% (two of 10) to CLA. They concluded that one individual patient can be colonized by multiple strains and that in individual patients *H. pylori* strains isolated from various sites may have different antibiotic susceptibilities.^23^ The study published by our group showed that 12% of Slovenian children were infected with more than one strain, determined by genetic testing.^24^ Therefore, a better eradication rate was probably achievable in our study by obtaining multiple specimens from different parts of the stomach for cultivation.

The success rate could also be influenced by duration of treatment. Data regarding the optimal duration of therapy for *H. pylori* eradication are conflicting. A meta‐analysis performed by Ford et al. tried to define the optimum duration of PPI‐based triple therapy among adults. Studies comparing 7‐day with 14‐day treatment with an identical drug combination showed that eradication rates were higher with longer duration of therapy. They concluded that treatment duration should be tailored according to the clinical situation.^25^ The authors of the Cochrane review, which included 75 studies, concluded that increasing the duration of PPI‐based triple therapy increases eradication rates among adults.^26^ The results in adults were not confirmed by the systematic review of *H. pylori* eradication treatment schedules in children published by Oderda et al.^27^She concluded that longer courses of PPI‐based triple therapies are not more effective than shorter ones. Our study, limited by a small sample size, did not show any statistical significant difference in eradication rate when comparing 7‐ and 10‐ vs 14‐day treatment schemes (*P*=.62).

Our study has several limitations. It was a retrospective study with all of the inherent limitations of this design. It was difficult to obtain the eradication data for all of the patients included. Namely, almost one‐third of patients were lost to follow‐up. In addition, although it was a multicenter study, it does not completely represent the Slovenian pediatric population infected with *H. pylori* because we were not able to obtain data from all Slovenian hospitals. Furthermore, the overall eradication rate was based on different durations of therapy, different antibiotic regimens and varied follow‐up tests to assess *H. pylori* eradication.

In conclusion, this is the first study that reports primary resistance and eradication success of tailored triple therapy for *H. pylori* infection among the Slovenian pediatric population. High CLA (23.4%) and MET (20.2%) primary resistances were detected. Our data strongly support the fact that in countries with high prevalence of resistant *H. pylori* strains susceptibility testing prior to eradication treatment is essential. Although triple therapy tailored to antimicrobial susceptibility was highly successful in our study, an eradication rate greater than 90% was not achieved.

## DISCLOSURE OF INTERESTS

The authors have no competing of interests.
